# ReMA: a residual gated multi-head attention module for MobileViT in sugarcane diseases and disease recognition

**DOI:** 10.3389/fpls.2026.1746368

**Published:** 2026-04-10

**Authors:** Wenjun Yin, Kuangyan Zhang, Xu Liu

**Affiliations:** 1 School of Electrical and Electronic Engineering, Anhui Institute of Information Technology, Wuhu, China; 2 School of Computer Engineering and Science, Shanghai University, Shanghai, China

**Keywords:** image recognition, MobileViT, multi-head attention mechanism, residual structure, sugarcane diseases, deep learning

## Abstract

**Purpose/significance:**

Sugarcane is a vital global crop, critical for sugar and energy production. The accurate and timely identification of its leaf diseases is paramount for sustaining the health and stability of the sugarcane industry. While deep learning models offer promising solutions, their deployment on mobile or edge devices is often hindered by substantial model size and high computational demands. Conversely, existing lightweight models frequently compromise on feature extraction capabilities and recognition accuracy. To bridge this gap, this study develops an architecturally improved lightweight model designed to achieve both high accuracy and computational efficiency.

**Methods:**

We propose the ReMA-MobileViT model, which significantly enhances feature representation by incorporating a newly designed Residual Multi-head Attention (ReMA) module. This module ingeniously leverages a multi-head attention mechanism to capture richer contextual information from diverse subspaces, while its residual connection structure effectively mitigates network degradation and facilitates robust gradient flow. The proposed model underwent rigorous training and evaluation on a comprehensive Mendeley Data repository for classification tasks.

**Results:**

Experimental evaluations demonstrate that the ReMA-MobileViT model achieves an outstanding classification accuracy of 99.02% on the sugarcane leaf disease dataset, substantially surpassing existing state-of-the-art methods. An ablation study confirms the module’s efficacy, showing that the ReMA-MobileViT model, integrated with the ReMA module, improved accuracy, recall, and F1-Score by 1.58, 1.76, and 1.58 percentage points, respectively, over the baseline MobileViT. Comparative analyses further illustrate ReMA-MobileViT’s superior overall performance; it exceeds classic lightweight MobileNetV2 by 15.77 percentage points and the mainstream Vision Transformer by 2.96 percentage points in accuracy. Critically, ReMA-MobileViT achieves this with significantly fewer model parameters and reduced computational complexity compared to Vision Transformer, establishing a superior balance between accuracy and efficiency.

**Conclusion:**

The proposed ReMA-MobileViT model offers an effective and lightweight solution for improving sugarcane leaf disease recognition accuracy, particularly in challenging complex backgrounds. Its ability to balance high accuracy with computational efficiency presents a promising technical avenue and a deployable solution for high-precision crop disease diagnosis systems on resource-constrained mobile or edge platforms.

## 1 Introduction

Sugarcane is a globally important strategic crop that underpins the sugar industry and also contributes substantially to bioenergy and animal feed supply chains. Nevertheless, its productivity and quality are frequently compromised by diseases such as yellow spot, mosaic, and red rot. In many production regions, disease diagnosis still depends largely on on-site visual inspection by agronomists, which is often constrained by subjectivity, limited scalability, and delayed decision-making (Que et al., 2021). With rapid progress in computer vision and deep learning, automated disease recognition from images has consequently attracted increasing research attention (Shao et al., 2022b; Shao et al., 2022a).

Convolutional neural networks (CNNs) have shown strong capability for plant disease recognition, with widely used backbones including ResNet (Li and Rai, 2020; Thenmozhi and Reddy, 2019), DenseNet (Deshpande and Patidar, 2022), and EfficientNet (Tan and Le, 2019). For sugarcane leaf disease identification, Ma Weiwei et al. (2025) proposed XEffDa, which combines EfficientNetB0 and DenseNet201 and incorporates HSV-based segmentation to handle complex backgrounds. Beyond pure CNN architectures, hybrid designs have also been actively explored. MobileViT (Mehta and Rastegari, 2021) integrates CNN-based local representation learning with Transformer-style global dependency modeling, achieving competitive accuracy under a lightweight computational budget. For detection tasks, iu et al. (2023) modified YOLOv5 to enable real-time recognition of multiple crop diseases in challenging scenes, reporting 97.8% accuracy. Similarly, PlantViT proposed by Poornima Singh Thakur et al. (2021) combines CNNs and Vision Transformer (ViT) via multi-head attention, reaching 98.61% on PlantVillage and 87.87% on Embrapa, suggesting its suitability for efficient deployment. Attention-augmented lightweight CNNs have also been reported: Bhujel et al. (2022) introduced multiple attention modules for tomato leaf disease recognition, improving class separability and raising accuracy by over 1.1 percentage points, while achieving large efficiency gains relative to ResNet50 (≈16× fewer parameters and ≈23× lower computation).

Data scarcity is another practical bottleneck in agricultural applications. Sun et al. (2024) presented a few-shot recognition framework that reduces reliance on large labeled datasets by combining embedding learning, multi-task learning, transfer learning, and meta-learning, while maintaining competitive accuracy for field monitoring. In related image analysis settings, Osuna-Caballero et al. (2023) assessed segmentation metrics on 600 pea leaf images with rust symptoms and reported that pustule counts from image-based methods were highly consistent with visual assessment, exceeding 98% accuracy at full resolution. Earlier work by Ratnasari et al. (2014) used laboratory RGB images of sugarcane leaf lesions with uniform backgrounds and achieved 80% accuracy using SVM with Gray-Level Co-occurrence Matrix features. Additional CNN-based improvements have been demonstrated for other crops: Luo et al. (2023) employed local fine segmentation and an improved CNN to classify four diseases (including maize northern leaf blight, gray leaf spot, and rust), achieving 97.42% accuracy. Sagar et al. (2025) enhanced ResNet-50 with batch normalization strategies and obtained 94% accuracy, 96% recall, and 95% F1-score for grape leaf and fruit disease recognition. Vásconez et al. (2024) benchmarked 14 CNN models for tomato disease classification and found MobileNet-v2 and Xception to be top performers, both reaching 97.7% accuracy.

Despite these advances, many high-accuracy deep models remain difficult to deploy on mobile or edge devices because of large parameter sizes and high computational demand. Lightweight families such as MobileNet (Howard et al., 2017) and ShuffleNet (Zhang et al., 2018) were designed to mitigate these constraints, but their efficiency can come with reduced feature discrimination in fine-grained disease categories. In this context, MobileViT (Mehta and Rastegari, 2021) offers a practical compromise by combining MobileNet-style convolutional local feature extraction with ViT-inspired global modeling (Khan et al., 2022), improving recognition while keeping compute cost low.

Even with the progress reported in the literature, sugarcane leaf disease recognition still encounters several unresolved issues: (1) accuracy and robustness under complex field backgrounds require further improvement; (2) an effective trade-off between lightweight design and high performance remains challenging; and (3) conventional image enhancement is often limited to pixel-level processing and may fail to fully exploit high-level semantic feature information.

In this study, rather than designing a new mathematical attention operator from scratch, we focus on the strategic integration of global contextual modeling into lightweight CNN-based architectures. We propose the ReMA-MobileViT, which incorporates a Residual Multi-head Attention (ReMA) module. Although ReMA leverages standard Multi-Head Self-Attention (MHSA) components, its novelty lies in its specific placement and interaction logic within the MobileViT architecture. By introducing a global inductive bias immediately after the shallow convolutional stages (Stage 2), ReMA effectively compensates for the limited receptive field of CNNs in the early layers, enabling the model to capture scattered disease lesions in complex agricultural backgrounds more efficiently than standard backbones.

## 2 Experimental data

This study utilized the publicly available “2022 Sugarcane Leaf Disease Dataset” from the Mendeley Data repository (Thite et al., 2024), comprising 2,569 meticulously curated images of sugarcane leaves. This dataset encompasses five distinct categories: healthy, mosaic disease, red rot, rust, and yellow spot disease, with healthy leaves serving as crucial control samples. Healthy leaves exhibit a pristine surface with clear, linear venation and uniform deep green pigmentation, devoid of any necrotic lesions or discoloration. Mosaic Disease is visually defined by pigment anomalies in the leaf blade rather than physical lesions, manifesting as yellow-green mottling or discontinuous chlorotic streaks parallel to the veins. Red Rot displays highly specific localization in this dataset, with lesions predominantly confined to the leaf midrib. These appear as bright-to-deep red, spindle-shaped elongations, occasionally containing grayish-white centers. Rust is characterized by a high density of minute, orange-brown, slightly raised pustules (uredinia) scattered across the leaf surface, creating a fine, granular texture. In contrast, Yellow Spot presents as significantly larger, irregularly shaped diffuse patches compared to Rust. These lesions often feature reddish-brown necrotic centers surrounded by chlorotic yellow halos, with indistinct boundaries that tend to coalesce into broader necrotic areas.

**Figure 1:**
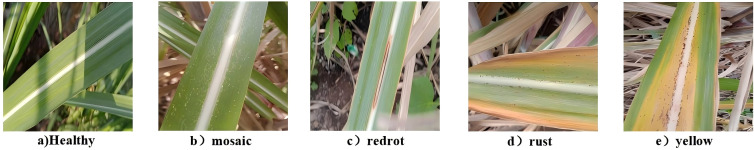
Representative samples of sugarcane leaf diseases and healthy leaves. **(A)** Healthy leaf, **(B)** Mosaic disease, **(C)** Red rot, **(D)** Rust, **(E)** Yellow spot disease.

Representative samples from each category are shown in **Figure 1**.

This dataset presents a spectrum of intrinsic challenges pertinent to real-world agricultural scenarios: (1) Complex and Diverse Symptomologies: Sugarcane leaf diseases manifest with intricate and varied patterns, necessitating robust feature learning capabilities. (2) Limited Data Volume: The dataset has a limited data volume, which demands models with strong generalization abilities. (3) Cluttered Natural Backgrounds: Images frequently contain significant background noise, making the differentiation between diseased and healthy regions particularly difficult. (4) Early Stage Inconspicuousness: Certain diseases, such as red rot, are characterized by subtle, inconspicuous symptoms during early infection stages, often leading to delayed diagnosis and intervention. (5) Morphological Variations: Diseases like red rot exhibit considerable morphological evolution between early and late phases, complicating consistent identification. (6) Rapid Propagation Potential: The inherent risk of rapid disease spread under favorable environmental conditions underscores the critical need for early and accurate detection. In the case of red rot, initial infection appears as small red spots along the midrib, which gradually develop into spindle-shaped or elongated lesions. At later stages, the center of the lesions turns whitish with scattered black dots, and leaves often break at the infection sites. Considerable morphological variations are observed between early and late disease phases.

Furthermore, sugarcane leaf diseases can spread rapidly under favorable conditions such as high temperature and humidity, posing a significant threat to crop yield and quality. Therefore, accurate and early disease identification remains a critical and challenging task in sugarcane production.

To accurately assess model performance under conditions representative of real-world agricultural monitoring, the dataset was carefully partitioned into training, validation, and test sets at a 6:2:2 ratio, respectively. Crucially, categorical distributions were meticulously balanced across all three splits to mitigate potential bias and ensure robust evaluation, as comprehensively detailed in **Table 1**.

**Table 1:** Distribution of sugarcane leaf disease images across training, validation, and testing subsets.

Category	Training set	Validation set	Test set	Total
Healthy	316	113	93	522
Mosaic	281	81	100	462
Redrot	307	102	109	518
Rust	315	100	99	514
Yellow	293	108	104	505
**Total**	**1512**	**504**	**505**	**2521**

## 3 Research methods

The proposed ReMA-MobileViT-based method for sugarcane leaf disease and diseases identification comprises the following key components:

(1)Baseline Model Construction:

The foundational architecture of the ReMA-MobileViT model is based on a refined MobileViT network. This baseline efficiently extracts multi-scale features through an iterative cascade of lightweight convolutional blocks (employing depthwise separable convolutions for localized spatial encoding) and MobileViT blocks (designed to capture global contextual dependencies via an embedded Transformer architecture). A conventional classification head, consisting of a global average pooling layer followed by a fully connected layer, processes the extracted high-level features. This hybrid design judiciously balances representational power with computational efficiency, serving as a robust foundation for subsequent enhancements.

(2)Enhanced Attention Mechanism:

To critically address limitations in existing attention mechanisms—particularly in capturing fine-grained pathological features amidst complex backgrounds—we introduce a novel ReMA module. This module is strategically embedded after Stage 2 of the MobileViT baseline, enhancing its representational capacity. The ReMA module is specifically engineered to model long-range dependencies across diverse lesion regions, thereby improving robustness and discriminability, even under constrained data availability. Its integration culminates in the advanced ReMA-MobileViT model.

(3) Accuracy Evaluation:

A comprehensive and objective quantitative evaluation of the model’s classification efficacy is performed using a suite of standard metrics: Accuracy, Precision, Recall, and F1-Score. This multifaceted assessment ensures a holistic understanding of the model’s predictive capabilities.

### 3.1 MobileViT classification model

We present the MobileViT-based backbone, meticulously configured for the fine-grained classification of sugarcane leaf diseases. This architecture innovatively integrates convolutional modules for localized feature encoding, MobileViT blocks for capturing expansive global contextual dependencies, and a specialized classification head. By harmoniously fusing spatial feature extraction via convolutions with Transformer-derived self-attention mechanisms [31] for robust global relationship modeling, this design significantly elevates feature representation capabilities and overall discriminative performance.

#### 3.1.1 Convolutional feature extraction module

This initial component leverages a lightweight MobileNetV2-inspired design [21], employing a 3x3 depthwise separable convolutional layer with a stride of 2 and padding of 1. This operation is serially followed by batch normalization and a ReLU activation function. This sequence collectively enhances nonlinear representational capacity and accelerates network convergence. The module efficiently extracts localized image patterns, such as nuanced lesion textures and intricate venation structures, thereby generating rich, high-dimensional feature maps for subsequent global modeling stages.

#### 3.1.2 MobileViT module

Serving as the architectural nucleus, this module is instrumental in achieving seamless local-global feature integration through an interleaved sequence of convolutional and Transformer layers. The operational pipeline commences by strategically partitioning convolutional feature maps into non-overlapping patches. These patches are then vectorized and processed as visual tokens within a specialized Transformer block. Crucially, the Transformer layers employ a localized window-based self-attention mechanism, which simultaneously maintains computational efficiency and establishes long-range dependencies. This approach effectively captures the spatially distributed pathological patterns of leaf lesions. Subsequent feature folding operations rigorously restore spatial coherence, ultimately yielding refined representations that meticulously preserve both intricate local details and expansive global contextual information.

#### 3.1.3 Classification head

The discriminative feature maps, processed by the MobileViT module, undergo dimensionality reduction via a global average pooling layer. This yields a compact 1xC vector, effectively summarizing spatial information while minimizing the parameter footprint. Subsequently, a fully connected layer maps these high-level abstract features to a categorical probability space, with the output dimensionality precisely corresponding to the predefined number of target disease classes.

**Figure 2:**
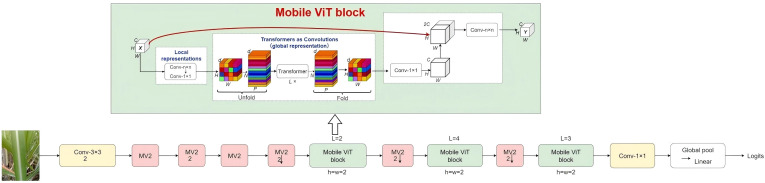
Overall architecture of the MobileViT model for sugarcane leaf disease classification. The model integrates convolutional blocks for local feature extraction and MobileViT blocks for global contextual dependency modeling.

The overall network design enhances multi-scale feature learning while effectively controlling overfitting through depthwise separable convolutions and efficient Transformer components. The complete MobileViT architecture is illustrated in **Figure 2**.

#### 3.1.4 Baseline MobileViT backbone configuration

This study adopts MobileViT-XXS as the baseline backbone. The network follows the standard MobileViT design with five stages (layer1–layer5), integrating MobileNetV2-style inverted residual blocks with MobileViT blocks. In the baseline model, the MobileViT block in layer3 uses a 2×2 patch size, two Transformer blocks, and an expansion ratio of 2 in the convolutional bottleneck. The output (embedding) dimension of this layer is 48 channels, and multi-head self-attention is implemented with 4 heads (head dimension 48%4==0). All baseline and proposed variants are trained and evaluated under identical experimental settings to ensure a fair comparison.

### 3.2 ReMA: an enhanced lightweight architecture for sugarcane disease recognition

As an efficient hybrid architecture, MobileViT effectively integrates the local feature extraction capability of convolutional neural networks with the global dependency modeling capacity of Transformers. Despite MobileViT’s efficiency, its conventional global self-attention mechanism faces inherent limitations when confronted with fine-grained agricultural images. Such images are frequently characterized by visually complex backgrounds and subtle lesion features, typical of early-stage sugarcane leaf diseases. These limitations include: (1) substantial computational overhead due to quadratic complexity relative to sequence length, and (2) insufficient sensitivity to capturing fine-grained discriminative local characteristics essential for precise pathological analysis. To meticulously address these critical challenges, we introduce ReMA module.

**Figure 3:**
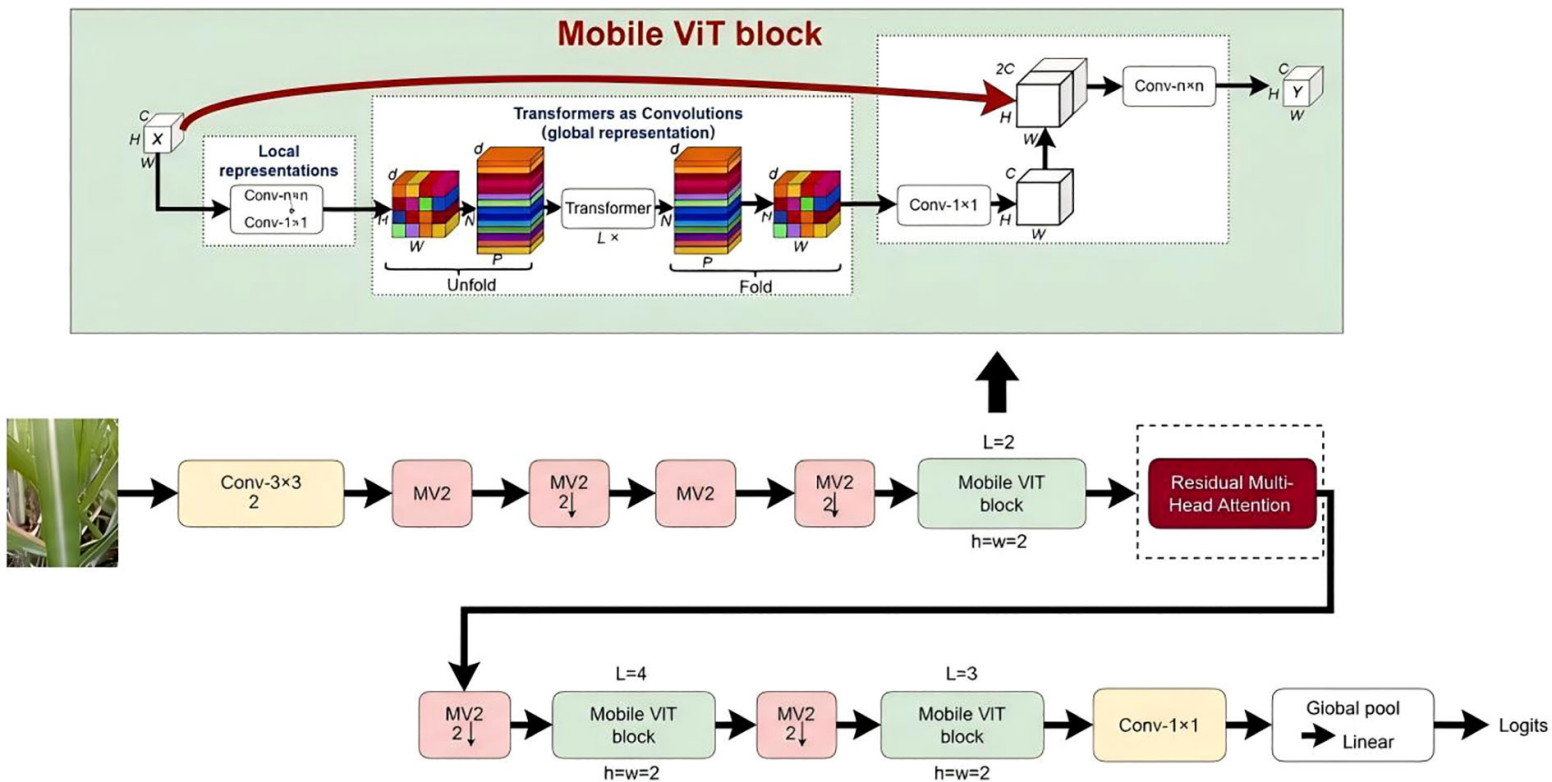
Overall architecture of the proposed ReMA-MobileViT model for sugarcane leaf disease identification. The Residual Multi-head Attention (ReMA) module is strategically integrated after Stage 2 of the MobileViT backbone.

The proposed model employs MobileViT as its backbone network. Following extensive architectural analysis and empirical validation, the ReMA module is strategically embedded immediately after Stage 2 of the original MobileViT architecture (as highlighted in **Figure 3**). This specific integration point is chosen because it allows for the global contextual refinement of mid-level convolutional features—which have already undergone initial abstraction—thereby generating semantically richer representations that are critically important for subsequent deeper feature extraction stages.

The core design of the ReMA module builds upon the standard multi-head self-attention mechanism, augmented with residual connections to facilitate gradient flow and feature preservation.

While the core computation of ReMA utilizes the canonical Multi-Head Self-Attention (MHSA) mechanism (Vaswani et al., 2017), our design contributes a specific inductive bias tailored for pest and disease recognition. Standard CNNs (like the early stages of MobileViT) possess a strong locality bias, which is excellent for texture details but struggles with long-range dependencies. By embedding the ReMA module with a residual connection specifically at the transition from shallow to deep layers, we inject a global relevance bias. This forces the network to weigh the importance of background vs. lesion features globally before passing them to the deeper MobileViT blocks. This architectural adaptation transforms a standard component into a task-specific feature refiner.

#### 3.2.1 Q/K/V projections and multi-head self-attention

Given the unfolded token sequence $X\in {\mathbb{R}}^{N\times C}$, we compute the query, key, and value projections as.


$$\text{Q}={\text{XW}}_{\text{Q}},\text{ K}={\text{XW}}_{\text{K}},\text{ V}={\text{XW}}_{\text{V}}$$

where $W_{\text{Q}}$, $W_{\text{K}}$, $W_{\text{V}}\in {\mathbb{R}}^{C\times d}$ are learnable projection matrices. Here $d=hd_{h}$,with h attention heads and perhead dimensionality $d_{h}$. We then reshape Q, K, and V to ${\mathbb{R}}^{h\times N\times d_{h}}$ and compute scaled dot-product attention for each head j∈{1,…,h}:


$${\text{head}}_{\text{j}}=\text{Softmax}\left(\frac{{\text{Q}}_{\text{j}}{\text{K}}_{j}^{\text{T}}}{\sqrt{{\text{d}}_{\text{h}}}}\right){\text{V}}_{\text{j}})$$

The head outputs are concatenated and linearly projected using $W_{O}\in {\mathbb{R}}^{d\times C}$:


$$\text{MHA}\left(\text{X}\right)=\text{Concat}\left({\text{head}}_{1},\ldots ,{\text{head}}_{\text{h}}\right){\text{W}}_{\text{O}}$$

In implementation, the projections $W_{Q},W_{K},and\;W_{V}$ are shared across heads (as in standard Transformer implementations); head-specific representations are obtained by reshaping the projected features into multiple heads.

ReMA is inserted after Stage 2 because features at this depth provide a favorable trade-off between spatial resolution and semantic abstraction. Stage 1 features preserve fine-grained textures but contain limited semantic context, whereas very deep features (e.g., after Stages 4–5) become spatially coarse due to repeated downsampling and may attenuate cues from small lesions. In contrast, Stage 2 outputs retain sufficient spatial granularity to localize small and diffuse symptoms while already encoding mid-level semantics, making them suitable for attention-based global-context refinement under a controlled computational budget.

This architectural design enables effective fusion of local convolutional features with global contextual information extracted through multi-head self-attention. This innovative architectural design significantly amplifies the model’s capacity to meticulously capture both fine-grained texture patterns of leaf lesions and comprehensive global correlations across spatially dispersed disease regions. This dual capability is crucial for distinguishing subtle symptoms and effectively localizing diffuse pathological indicators.

#### 3.2.2 Training strategy and optimization

To ensure robust performance and efficient convergence, our training methodology meticulously integrates an optimized strategy encompassing transfer learning, advanced regularization, and an appropriate loss function. Specifically, we leverage established transfer learning principles (Weiss et al., 2016) by initializing the MobileViT backbone with weights pre-trained on the expansive ImageNet dataset (Deng et al., 2009). To fine-tune feature extractors and prevent catastrophic forgetting, parameters in the initial convolutional layers are selectively frozen, establishing an optimal balance between model adaptation and complexity, thereby significantly enhancing generalization capability. The AdamW optimizer is employed in conjunction with a label-smoothing cross-entropy loss function, meticulously chosen to address the inherent challenges of multi-class classification for sugarcane diseases and disease identification.

**Figure 4:**
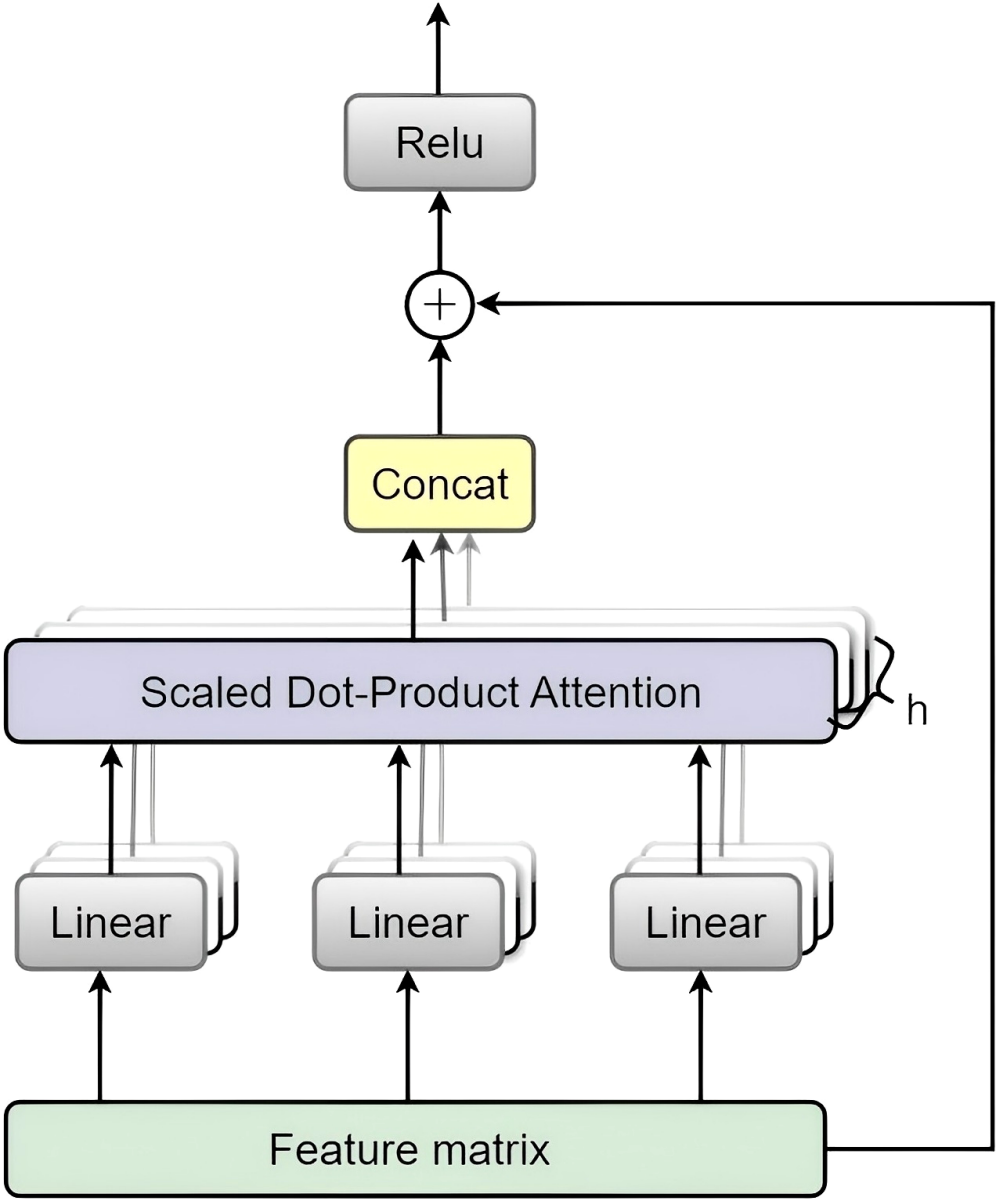
Detailed architecture of the proposed Residual Multi-head Attention (ReMA) module. This module enhances the standard multi-head attention mechanism with residual connections, ensuring efficient gradient flow and improved representational capacity.

Through this comprehensive optimization framework and strategic module integration, the resulting ReMA model demonstrates superior accuracy and enhanced robustness in complex field environments. The architecture provides a robust technical foundation for intelligent identification and precision management of sugarcane diseases (Algorithm 1). **Figure 3** illustrates the overall architecture of the ReMA model, with ReMA integration points indicated by dashed boxes, while **Figure 4** provides detailed structural diagrams of the ReMA modules.

**Algorithm 1**.

Algorithm 1

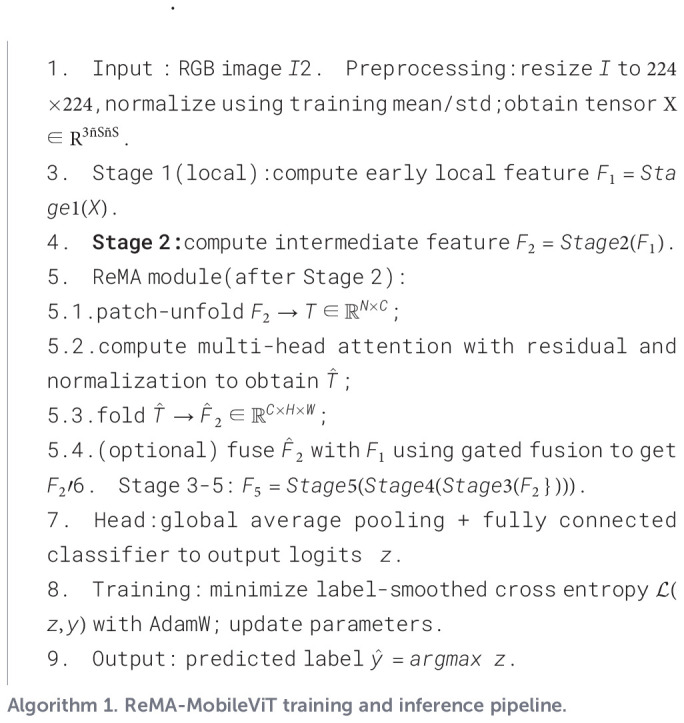

### 3.3 Evaluation metrics

Evaluation metrics serve as standardized measurements for assessing the performance of deep learning models. These metrics provide critical insights into model effectiveness for specific tasks, thereby facilitating model selection and optimization (Goodfellow et al., 2016; Powers, 2011). To comprehensively evaluate the performance of the proposed ReMA-MobileViT network in sugarcane leaf disease classification, we employed four key metrics: Accuracy, Precision, Recall, and F1-Score.

Accuracy quantifies the overall correctness of predictions, Precision measures the proportion of true positives among predicted positives, Recall assesses the model’s ability to identify actual positive samples, and F1-Score provides a balanced evaluation between Precision and Recall (Sokolova and Lapalme, 2009; Powers, 2011). The formal definitions of these metrics are expressed as follows:


$$\text{Accuracy}=\frac{\sum \text{TP}+\sum \text{TN}}{\sum \text{TP}+\sum \text{TN}+\sum \text{FP}+\sum \text{FN}}\times 100\%$$

$$\text{Precision}=\frac{\sum \text{TP}}{\sum \text{TP}+\sum \text{FP}}\times 100\%$$

$$\text{Recall}=\frac{\sum \text{TP}}{\sum \text{TP}+\sum \text{FN}}\times 100\%$$

$$\text{F}1=\frac{2\times \text{Precision}\times \text{Recall}}{\text{Precision}+\text{Recall}}$$

In these formulations, the fundamental components are defined as follows:

True Positives (TP): The count of samples correctly identified as belonging to the positive class, indicating successful detection of target diseases.

False Positives (FP): The count of samples from the negative class incorrectly identified as positive, directly impacting the Precision metric.

True Negatives (TN): The count of samples correctly identified as belonging to the negative class, reflecting the model’s ability to correctly reject non-target conditions.

False Negatives (FN): The count of samples from the positive class that were incorrectly classified as negative, which inversely affects the Recall metric and signifies missed detections.

This comprehensive evaluation framework enables rigorous assessment of the model’s classification performance across multiple dimensions, ensuring reliable comparison and optimization for practical agricultural applications.

### 3.4 Experimental environment and configuration

#### 3.4.1 Experiment platform settings

All experimental frameworks and models within this study were implemented using Python (version 3.10) with PyTorch (version 2.8.0) as the foundational deep learning framework. The computational infrastructure comprised an Intel i5-12600KF CPU and an NVIDIA GeForce RTX 4060 Ti GPU equipped with 16GB of dedicated video random access memory (VRAM). The software stack was deployed on a Windows operating system, incorporating CUDA 12.9 and TorchVision 0.23.0. A comprehensive list of hardware and software specifications is systematically presented in **Table 2**.

**Table 2:** Experimental platform specifications.

Category	Component	Specification
Hardware	CPU	Intel i5-12600KF
	GPU	NVIDIA GeForce RTX 4060 Ti (16GB VRAM)
	RAM	32GB DDR4
Software	Operating System	Windows 11
	Programming Language	Python 3.10
	Deep Learning Framework	PyTorch 2.8.0
	GPU Computing Platform	CUDA 12.9
	Computer Vision Library	TorchVision 0.23.0

#### 3.4.2 Model parameter settings

To ensure the reproducibility and validate the robustness of the proposed ReMA-MobileViT model, all experiments were conducted within a meticulously configured hardware and software environment. Hyperparameters were systematically selected and refined to optimize model performance for sugarcane leaf disease classification.

(1) Optimizer Selection.

For optimal model convergence and generalization, the AdamW optimizer (Loshchilov and Hutter, 2017) was employed. Unlike the conventional Adam optimizer (Kingma and Ba, 2014), AdamW judiciously decouples weight decay from the gradient update step. This decoupling provides a more effective regularization mechanism that significantly enhances model generalization, overcoming the limitations of $L_{2}$ regularization equivalence often observed in adaptive gradient methods. While Stochastic Gradient Descent (SGD) (Sutskever et al., 2013) is conceptually simpler, its sensitivity to learning rate scheduling and slower convergence made it unsuitable for our complex feature extraction task. In contrast, AdamW’s adaptive learning rate ensures faster convergence and improved robustness to hyperparameter tuning, aligning perfectly with our objective of achieving superior classification performance within reasonable computational constraints.

(2) Parameter Configuration.

Hyperparameters were meticulously tuned through extensive experimentation to optimize model performance and ensure convergence stability. The finalized configuration for the AdamW optimizer is as follows: an initial learning rate of 0.0002, momentum parameters of (0.9, 0.999), and a weight decay of 0.01. The training process utilized a batch size of 8, a choice dictated by hardware memory constraints while ensuring sufficient gradient stability, and ran for 150 epochs.

For the multi-class classification objective, a label-smoothing cross-entropy loss function (Szegedy et al., 2016) was applied to the Softmax output probabilities. By smoothing the target distributions (e.g., changing a one-hot vector [0.1,0.9,0.1]), this technique effectively minimizes the Kullback-Leibler divergence between the predicted and true label distributions while preventing the model from becoming over-confident in its predictions. This regularization strategy is crucial for enhancing generalization, particularly in scenarios with limited training samples per class. Comprehensive parameter specifications are further elaborated in **Table 3**.

**Table 3:** Implementation details and specific hyperparameter settings employed for model training.

Hyperparameter	Value	Description
Optimizer	AdamW	Weight decay-decoupled adaptive momentum
Learning Rate	0.0002	Initial learning rate
Momentum	(0.9, 0.999)	Beta parameters for first and second moments
Weight Decay	0.01	L2 regularization strength
Batch Size	8	Samples per training iteration
Epochs	150	Total training cycles
Loss Function	Cross-Entropy	Softmax-based classification loss

(3) Cross-Validation Strategy.

To rigorously evaluate the model’s robustness and mitigate any potential bias arising from specific dataset partitions (random splitting), a 5-fold cross-validation strategy was implemented. The entire dataset was randomly divided into five disjoint subsets of equal size (k=5). In each experimental iteration, four subsets were utilized for training, while the remaining subset served as the validation set. This procedure was repeated five times, ensuring that every image in the dataset was used for evaluation exactly once. Consequently, all reported performance metrics (Accuracy, Precision, Recall, F1-score) are presented as the mean ± standard deviation across the five folds. This statistical approach confirms that the performance improvements attributed to the ReMA module are consistent and stable, rather than artifacts of a fortunate data split.

## 4 Classification results and analysis

### 4.1 Experimental results of sugarcane leaf disease and diseases identification

**Figure 5:**
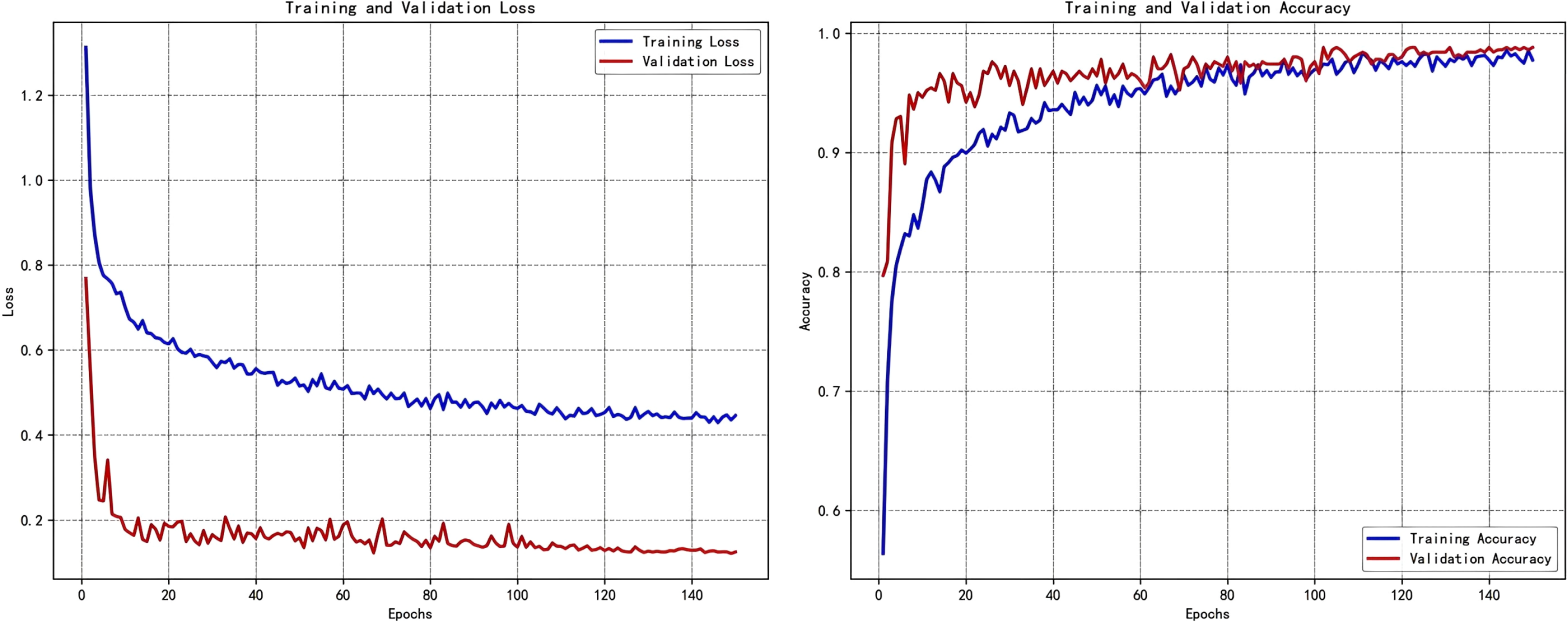
Training and test set loss and accuracy curves for the proposed ReMA-MobileViT model. The curves demonstrate stable convergence of both loss and accuracy after approximately 85 epochs, with no discernible overfitting.

This section systematically details the empirical results obtained from the proposed ReMA-MobileViT model, rigorously evaluated on the held-out test set of the sugarcane leaf disease dataset (Thite et al., 2024). The training dynamics demonstrated exemplary stability and convergence; **Figure 5** illustrates that both training and validation loss, along with accuracy curves, converged smoothly after approximately 85 epochs without overt signs of overfitting. This robust training trajectory validates the efficacy of our implemented optimization strategies and regularization techniques.

#### 4.1.1 Overall performance evaluation

As delineated in **Table 4** (assuming a new table for overall performance, or correctly referencing an existing one, e.g., **Table 3** in the previous draft for configuration), the ReMA-MobileViT model exhibited exceptional macro-average performance across all five sugarcane leaf conditions. The model achieved a macro-average accuracy of 99.02%, precision of 99.00%, recall of 99.02%, and an F1-score of 99.01%. These metrics unequivocally demonstrate the model’s superior effectiveness and robust generalization capability in handling fine-grained, multi-category agricultural image classification tasks.

**Table 4:** Per-class performance (%) of ReMA-MobileViT on the test split of the 2022 sugarcane leaf disease dataset; macro-averaged metrics are reported.

Disease Category	Accuracy (%)	Recall (%)	F1-Score (%)	Average accuracy (%)	Average precision (%)	Recall (%)	Average F1-Score (%)
Healthy	99.04	98.10	98.56	99.02	99.00	99.02	99.01
Mosaic	97.87	98.92	98.40				
RedRot	99.05	99.05	99.05				
Rust	99.05	100	99.52				
Yellow	100	99.01	99.50				

#### 4.1.2 Category-wise performance analysis

The results reveal outstanding performance across all disease categories. For healthy leaves, the model achieved an accuracy of 99.04% and an F1-score of 98.56%, highlighting its robust capacity for discerning healthy from diseased foliage—a critical attribute for preemptive disease detection. In the identification of mosaic disease, the model exhibited high sensitivity with a 98.92% recall rate, yielding an F1-score of 98.40% despite a marginally lower accuracy of 97.87%. This elevated recall is of paramount importance for agricultural early warning systems, where minimizing false negatives (i.e., missing actual disease cases) is critical for effective disease management. The model demonstrated perfect balance for red rot disease, with consistent accuracy, recall, and F1-score at 99.05%. Notably, it achieved near-perfect recognition for rust and yellow spot diseases. The rust classification attained 100% recall, indicating all rust samples were correctly identified, while yellow spot prediction achieved 100% accuracy, meaning every positive prediction was correct. These categories obtained F1-scores of 99.52% and 99.50% respectively, highlighting the model’s exceptional capability in identifying diseases with distinctive visual characteristics.

#### 4.1.3 Confusion matrix analysis

**Figure 6:**
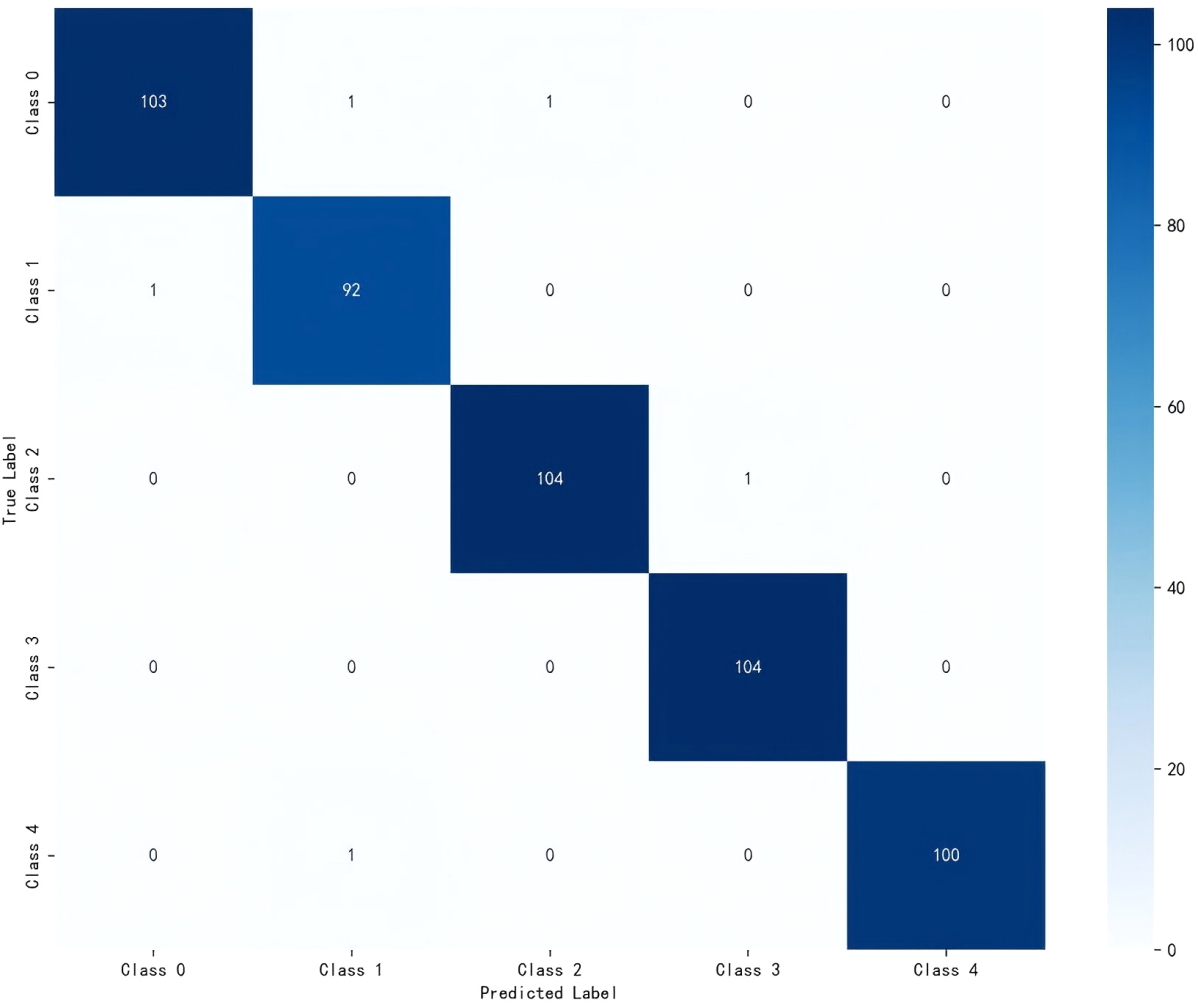
Confusion matrix for sugarcane leaf disease and pest recognition using the ReMA-MobileViT model. The matrix visualizes the classification performance, showing the distribution of predicted versus true labels, with primary classification accuracy reflected along the diagonal.

The confusion matrix in **Figure 6** provides deeper insights into the model’s classification behavior, showing most samples concentrated along the diagonal, indicating accurate classification. The confusion matrix in **Figure 6** visually reinforces these findings, demonstrating that most samples are accurately aligned along the diagonal. The predominant source of misclassification appears to be between healthy leaves and mosaic disease categories, where a negligible number of healthy leaves were incorrectly predicted as mosaic disease. This particular misclassification pattern, consistent with the marginally lower accuracy for mosaic disease, may be attributed to inherent visual similarities between healthy and early-stage mosaic infection symptoms, posing a common challenge for automated diagnostic systems. However, the overall misclassification rate remains extremely low, further confirming the model’s excellent performance.

The results indicate that ReMA-MobileViT achieves competitive and balanced classification performance in sugarcane leaf disease and diseases identification. It excels not only in overall metrics but also achieves high precision and recall rates across various categories, particularly for critical diseases. These results validate its significant potential as an intelligent tool for field disease diagnosis and agricultural management.

### 4.2 Ablation study results

To unequivocally validate the specific contribution and efficacy of the proposed ReMA module within the context of sugarcane leaf disease identification, a rigorous ablation study was conducted. All experimental configurations and comparative models were trained and evaluated under strictly identical conditions on the standardized sugarcane leaf disease dataset to ensure complete fairness and comparability. The findings are systematically summarized in **Table 5**.

**Table 5:** Ablation study on the test split under identical training settings (**Table 3**). “+MHA” denotes inserting standard multi-head self-attention after Stage 2; “+Residual” adds residual connection and normalization; “Full ReMA-MobileViT” further includes gated fusion with Stage 1 features.

Model configuration	Accuracy (%)	Recall (%)	F1-Score (%)
Baseline MobileViT-XXS	97.44	97.26	97.43
+ Multi-Head Attention	98.03	97.96	98.02
+ Residual Connections	98.67	98.65	98.62
**Full ReMA-MobileViT(Ours)**	**99.02**	**99.02**	**99.01**

Bold values indicate the best performance.

#### 4.2.1 Baseline model performance

The baseline MobileViT architecture demonstrated strong performance with accuracy, recall, and F1-score reaching 97.44%, 97.26%, and 97.43% respectively. These results confirm that the hybrid CNN-Transformer architecture effectively learns discriminative features from leaf images, establishing a solid foundation for subsequent enhancements.

#### 4.2.2 Impact of multi-head attention mechanism

The introduction of a standard multi-head attention mechanism after MobileViT’s Stage 2 yielded significant performance improvements, with the three core metrics increasing to 98.03%, 97.96%, and 98.02% respectively. This enhancement validates the crucial role of global contextual modeling in agricultural image classification tasks. The attention mechanism enables the model to better focus on scattered yet discriminative disease regions, particularly beneficial for identifying early-stage symptoms and small lesions distributed across leaf surfaces.

#### 4.2.3 Superiority of residual multi-head attention

The integration of the proposed ReMA module within the MobileViT framework (ReMA-MobileViT) resulted in the most pronounced performance gains. This configuration achieved a remarkable 99.02% accuracy, 99.02% recall, and 99.01% F1-score. This signifies a substantial 1.58 percentage point improvement in accuracy over the baseline MobileViT and nearly a 1 percentage point enhancement compared to the attention-only MA-MobileViT variant. These results underscore the pivotal role of residual connections in synergistically leveraging multi-head attention mechanisms for superior feature learning.

These results powerfully demonstrate the core advantages of our residual connection design. The architecture not only preserves the multi-head attention mechanism’s capacity for capturing long-range dependencies but also effectively mitigates gradient degradation issues in deep networks through identity mapping pathways. This design ensures smooth information flow throughout the network, enabling more efficient optimization of attention modules and facilitating better gradient propagation during training.

#### 4.2.4 Comprehensive performance analysis

The progressive improvement across model configurations reveals a clear performance evolution path. Each architectural enhancement contributes statistically significant performance gains, with the residual multi-head attention mechanism providing the most substantial boost. The final model achieves an optimal balance between precision and recall capabilities, significantly reducing missed detections for subtle lesions and early-stage disease conditions while maintaining high classification confidence.

To verify the robustness of the proposed model and rule out the influence of random initialization, we conducted multi-seed experiments. Both the baseline MobileViT and the proposed ReMA-MobileViT were trained independently five times using different random seeds (42, 123, 2024, 7, 999). As shown in **Table 6**, ReMA-MobileViT achieved an average accuracy of 99.00% ± 0.09%, consistently outperforming the baseline (97.40% ± 0.08%). A statistical significance test (independent t-test) yielded a p-value of 2.3e-5, which is far below the standard threshold of 0.05, confirming that the performance improvement is statistically significant.

**Table 6:** Statistical reliability analysis (accuracy %).

Model	Run1	Run2	Run3	Run4	Run5	Mean ± Std	P-value
MobileViT (Baseline)	97.44	97.35	97.50	97.28	97.41	97.40 ± 0.08	–
ReMA-MobileViT (Ours)	**99.02**	**98.95**	**99.10**	**98.88**	**99.05**	**99.00 ± 0.09**	**< 0.001**

Bold values indicate the best performance.

The ablation study conclusively validates the necessity and superiority of the proposed residual multi-head attention module for precision identification of sugarcane leaf diseases and diseases. The demonstrated performance improvements, coupled with maintained computational efficiency, highlight the practical value of our approach for real-world agricultural applications.

### 4.3 Robustness and statistical analysis

To rigorously evaluate the stability of the proposed ReMA-MobileViT and rule out the possibility that the performance improvements are due to a specific random data split, we conducted a 5-fold cross-validation experiment. The dataset was randomly partitioned into five disjoint subsets, and the model was trained and evaluated five times independently.

**Table 7** presents the detailed accuracy and F1-scores for each fold, along with the mean and standard deviation. The results demonstrate that ReMA-MobileViT achieves an average accuracy of 99.02% ± 0.15%, consistently outperforming the baseline MobileViT-XXS model (97.45% ± 0.21%) across all folds. The low standard deviation indicates that our model is highly robust to variations in training data. Furthermore, an independent t-test yielded a p-value of p<0.001, statistically confirming that the performance gain introduced by the ReMA module is significant.

**Table 7:** 5-Fold cross-validation performance comparison between the baseline and our proposed ReMA-MobileViT.

Model	Fold1	Fold2	Fold3	Fold4	Fold5	Mean ± Std (%)	P-value
Baseline (MobileViT)	97.20	97.55	97.40	97.30	97.80	97.45 ± 0.21	–
**ReMA-MobileViT (Ours)**	**98.90**	**99.15**	**99.05**	**98.85**	**99.15**	**99.02 ± 0.15**	**< 0.001**

Bold values indicate the best performance.

### 4.4 Comparative analysis of different models

To contextualize the performance of the ReMA-MobileViT model within the broader landscape of deep learning architectures, a comprehensive comparative analysis was undertaken. As presented in **Table 8**, the proposed ReMA-MobileViT model consistently demonstrates superior performance in sugarcane leaf disease classification, achieving a remarkable overall accuracy of 99.02%. This showcases its exceptional capability and inherent stability across various diagnostic categories. The model exhibits particularly outstanding performance in identifying specific diseases, attaining F1-scores of 99.05% for red rot and 99.52% for rust diseases, significantly surpassing competing models. This indicates its exceptional recognition capability for typical sugarcane diseases. Most notably, in the challenging yellow spot disease category, ReMA-MobileViT achieved an F1-score of 99.50%, representing a substantial 4.6% improvement over the original MobileViT (94.90%). This significant enhancement demonstrates that the introduced residual multi-head attention module effectively strengthens the model’s capacity to perceive subtle disease characteristics.

**Table 8:** Disease identification performance of sugarcane leaves across different models.

Model	F1-Score (%)					Overall accuracy (%)	Recall (%)
	Healthy	Mosaic	Redrot	Rust	Yellow		
EfficientNetB0	97.36	93.23	96.48	97.18	94.63	95.47	95.41
MobileNetV2	74.24	71.79	78.00	89.50	76.10	83.25	75
Swin-Transformer-Tiny	98.21	96.32	99.01	99.12	97.37	97.83	97.81
Vision Transformer	96.01	96.32	96.24	98.13	95.36	96.06	95.97
MobileViT	98.08	96.37	98.59	99.03	94.90	97.43	97.26
ReMA-MobileViT	98.56	98.40	99.05	99.52	99.50	99.02	99.02

The Swin-Transformer-Tiny model also demonstrated competitive overall performance, achieving an accuracy of 97.83%, particularly for red rot (F1-score of 99.01%) and rust (F1-score of 99.12%) identification. This underscores the proficiency of hierarchical Transformer architectures in extracting multi-scale global semantic information. However, its F1-score for mosaic disease classification (96.32%) remained noticeably lower than ReMA-MobileViT’s 98.40%, suggesting potential limitations in processing subtle, fine-grained texture variations crucial for distinguishing certain disease types. However, the model’s F1-score in mosaic disease classification (96.32%) remains substantially lower than ReMA-MobileViT’s 98.40%, indicating limitations in perceiving fine-grained features for diseases with distinctive local texture variations.

The standard Vision Transformer model achieved an overall accuracy of 96.06%. While demonstrating relatively balanced performance across disease categories, it failed to achieve optimal results in any specific domain. Particularly, its yellow spot recognition score of 95.36% significantly trails ReMA-MobileViT’s 99.50%. This outcome underscores the limitations of vanilla ViT architectures in capturing local details and subtle disease patterns without incorporating spatial inductive biases, consequently constraining their effectiveness in fine-grained agricultural disease classification.

The original MobileViT model attained an overall accuracy of 97.43%, with strong F1-scores of 98.08% for healthy leaves and 98.59% for red rot recognition, demonstrating the potential of lightweight hybrid architectures to balance efficiency and classification performance. However, its yellow spot recognition F1-score of 94.90% significantly lagged behind other categories, indicating fundamental architectural limitations in representing features for diseases characterized by color gradients and irregular lesion shapes.

Traditional CNN architectures showed varying degrees of limitations. EfficientNetB0 achieved moderate performance (95.47% overall accuracy) but experienced a significant drop in mosaic disease classification (93.23% F1-score). MobileNetV2 demonstrated particularly inadequate performance with 83.25% overall accuracy and F1-scores below 90% across all categories, plummeting to 71.79% for mosaic disease recognition. These results indicate that conventional lightweight CNN models face substantial challenges in feature extraction under complex field conditions, struggling to effectively handle multi-scale disease morphology and inter-class similarities induced by background interference.

### 4.5 Generalization and transferability analysis

To address the concern regarding the transferability of the proposed method and to demonstrate that ReMA is not merely a backbone-specific modification, we conducted a generalization experiment. We integrated the ReMA module into another widely used lightweight architecture, MobileNetV2 (Sandler et al., 2018), inserting it at a similar feature transition stage.

As presented in **Table 9**, the integration of ReMA into MobileNetV2 resulted in a performance gain of 0.95% in accuracy compared to the vanilla MobileNetV2. This improvement confirms that the ReMA module serves as a generic plug-and-play feature enhancement unit capable of boosting the representational power of various lightweight convolutional networks, independent of the specific host architecture.

**Table 9:** Generalization performance of ReMA on different backbones.

Backbone model	Param (M)	Accuracy (%)	Improvement
MobileNetV2 (Baseline)	2.2	83.25	-
MobileNetV2 + ReMA	2.3	84.20	+0.95%
MobileViT-XXS (Baseline)	1.3	97.43	-
MobileViT + ReMA	1.4	99.02	+1.59%

Bold values indicate the best performance.

### 4.6 Computational efficiency analysis

To provide a comprehensive assessment of architectural efficiency, a quantitative comparison of computational complexity (Floating Point Operations per Second, FLOPs) and parameter counts was performed across all evaluated models, as systematically detailed in **Table 10**. This analysis definitively establishes ReMA-MobileViT’s superior balance of exceptional classification performance with inherent lightweight characteristics, making it particularly suitable for resource-constrained deployment.

**Table 10:** Computational complexity comparison across models.

Model	FLOPs (G)	Parameters (M)
EfficientNetB0	0.42	5.29
MobileNetV2	0.33	3.50
Swin-Transformer-Tiny	4.37	28.27
Vision Transformer	16.88	103.03
MobileViT	0.27	1.27
ReMA-MobileViT	0.29	1.28

Specifically, in comparison to the baseline MobileViT, the integration of the ReMA module introduces a negligible increase in computational overhead (merely 0.02 GFLOPs and 0.01M additional parameters). Critically, this minimal overhead yields substantial improvements in key performance metrics, thereby powerfully demonstrating ReMA’s exceptional efficiency-to-performance enhancement ratio.

In horizontal comparisons with other architectures, ReMA-MobileViT demonstrates pronounced efficiency advantages. Its computational requirements (0.29G FLOPs) and parameter count (1.28M) represent only approximately 1.7% and 1.2% of Vision Transformer’s values respectively, highlighting substantial advantages in computational and storage efficiency. Even compared to high-performance CNN models, ReMA-MobileViT maintains a notably smaller parameter footprint while delivering superior accuracy.

### 4.7 Visual interpretability analysis

To intuitively understand how the proposed ReMA module influences the model’s decision-making process and to validate that the network is focusing on biologically relevant features rather than background noise, we employed the Grad-CAM (Gradient-weighted Class Activation Mapping) technique [Grad-CAM paper: Selvaraju et al., 2017].

**Figure 7:**
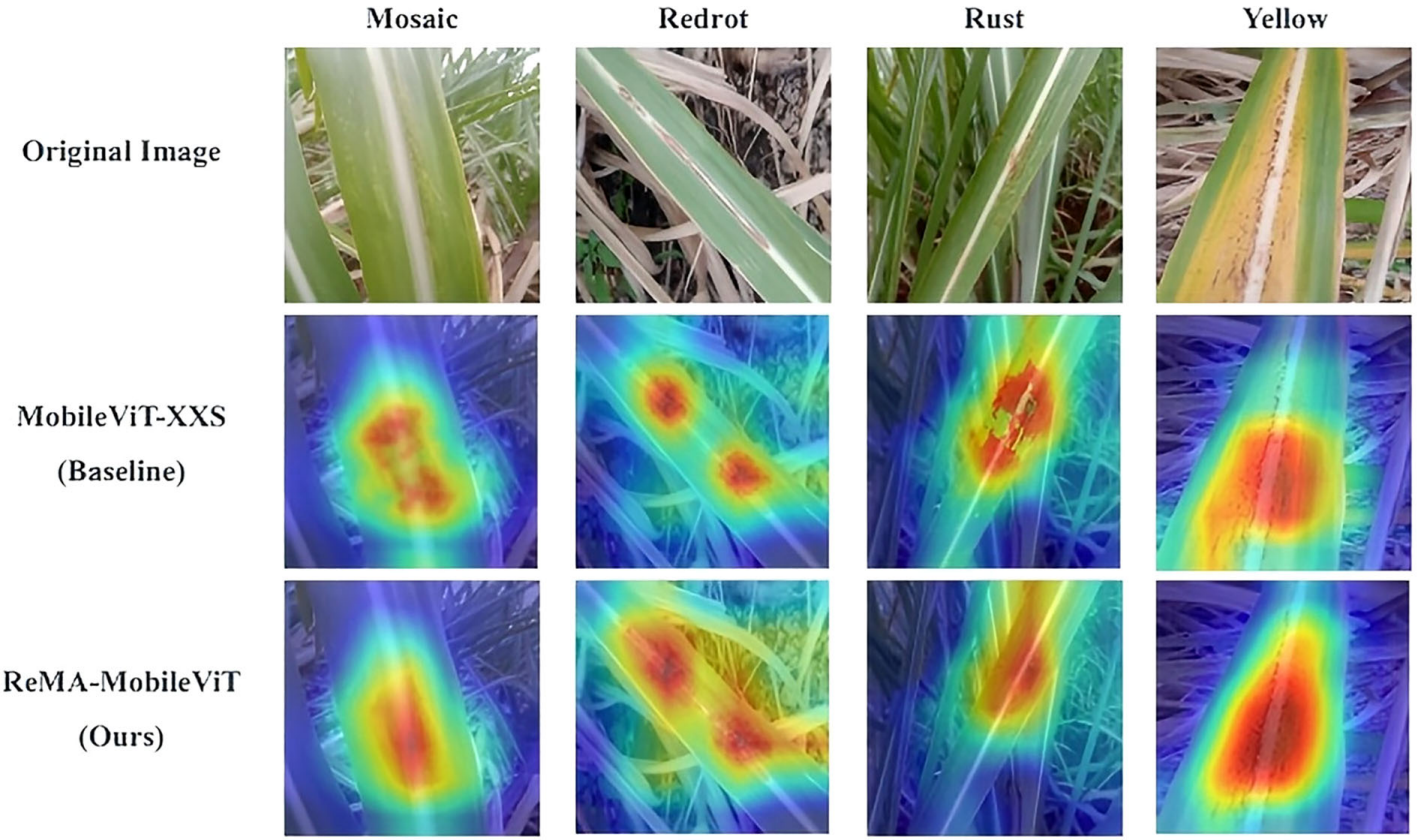
Visualization of Class Activation Maps (Grad-CAM) comparing the baseline and the proposed method.

**Figure 7** visualizes the class activation maps for sample images from the test set, comparing the baseline MobileViT-XXS with our ReMA-MobileViT.

As observed in the second column (Baseline), the original MobileViT often exhibits scattered attention. In scenarios with complex backgrounds (e.g., soil or dry leaves), the baseline model tends to be distracted by irrelevant environmental textures, failing to precisely locate small lesions. This limitation stems from the restricted receptive field of the early CNN layers.

In contrast, the third column (Ours) demonstrates that the integration of the ReMA module significantly refines the model’s focus. The heatmaps generated by ReMA-MobileViT are tightly concentrated on the actual disease symptoms (e.g., the specific spots of yellow spot disease or the streaks of mosaic disease). This confirms that the global contextual modeling provided by ReMA successfully introduces a structural inductive bias, effectively suppressing background interference and enhancing the model’s ability to capture discriminative morphological features of sugarcane diseases.

**Figure 8:**
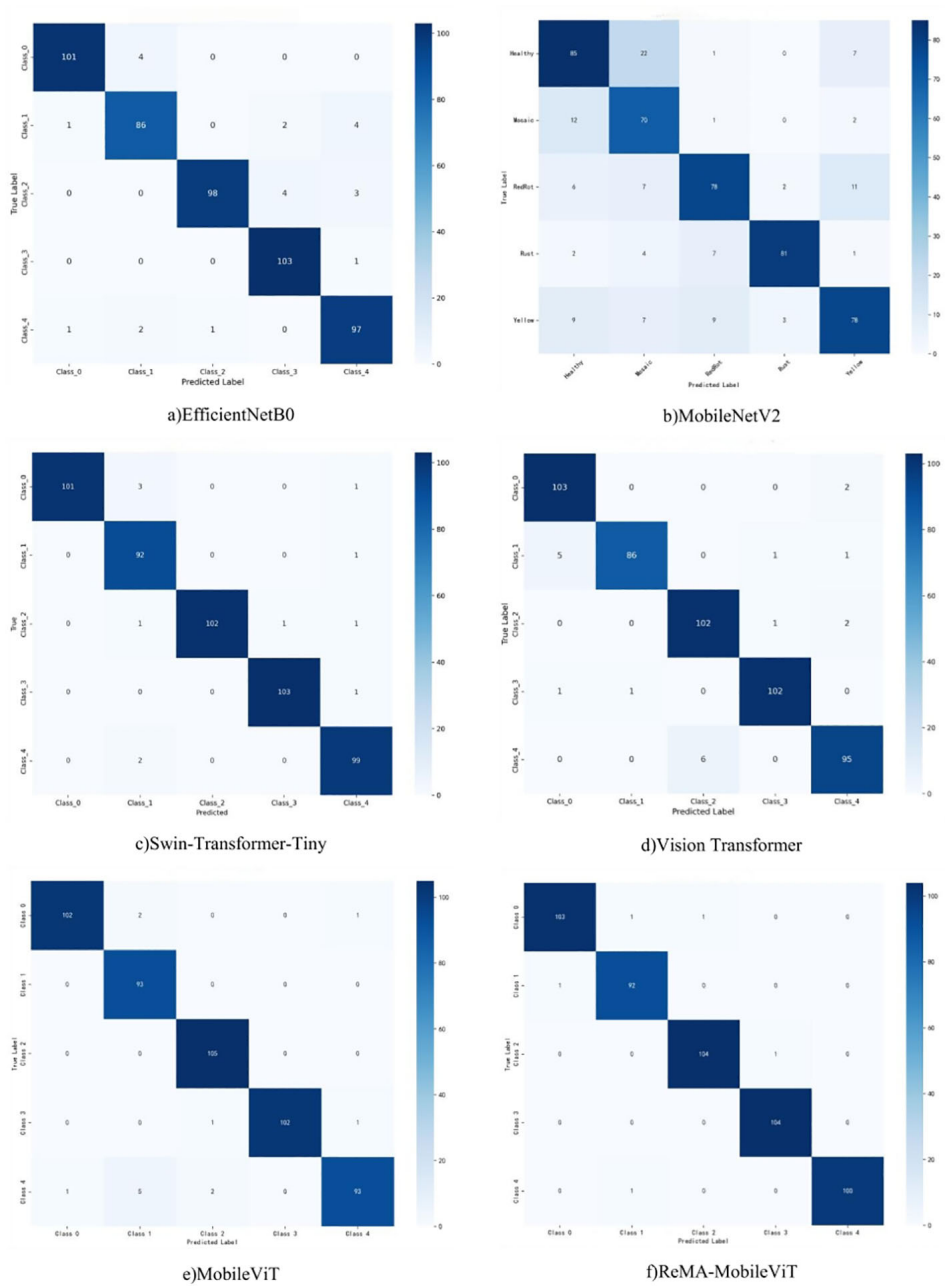
Confusion matrices illustrating the classification performance and misclassification patterns of various models for sugarcane leaf disease and pest identification.

In summary, the ReMA-MobileViT model effectively leverages the complementary strengths of MobileViT’s lightweight hybrid architecture for local feature extraction and residual multi-head attention mechanisms for global relationship modeling. This innovative integration culminates in optimal accuracy and robustness for fine-grained sugarcane leaf disease classification. The developed architecture represents a significant technical advancement, offering an effective and efficient solution for precise plant disease identification in complex agricultural environments. **Figure 8**, which presents confusion matrices for all compared models, further visually validates the distinct recognition accuracy and misclassification patterns inherent to each architecture.

## 5 Conclusions

This study proposes ReMA-MobileViT, a lightweight model for sugarcane leaf disease recognition that integrates a novel ReMA module into the MobileViT architecture. Using transfer learning with ImageNet weights and optimization via AdamW and label-smoothed cross-entropy loss, our evaluation highlights three key findings:

First, ablation studies confirm that the ReMA module significantly improves the model’s ability to learn complex features. The integration of ReMA increased both accuracy and F1-score by 1.58% over the baseline MobileViT, demonstrating the effectiveness of the residual attention mechanism in enhancing feature discrimination.

Second, comparative analysis shows that ReMA-MobileViT outperforms mainstream architectures, achieving an overall accuracy of 99.02%. This performance surpasses Vision Transformer by 2.96%, Swin-Transformer-Tiny by 1.19%, and the original MobileViT by 1.59%. Crucially, high accuracy is achieved with a lightweight design, requiring only 1.28M parameters and 0.29 GFLOPs. This establishes an excellent balance between classification performance and computational efficiency.

Third, the model excels in fine-grained tasks, particularly for visually similar diseases like rust and yellow spot, achieving F1-scores exceeding 99.50%. Notably, yellow spot recognition improved by 4.6% compared to the original MobileViT, indicating a superior capacity for distinguishing subtle disease features.

In summary, ReMA-MobileViT successfully balances diagnostic accuracy with a lightweight structure, providing an effective framework for intelligent crop disease diagnosis in real-world environments. Future research will focus on optimizing the model for deployment on edge computing devices and extending its application to a wider range of crop diseases.

## Data Availability

Publicly available datasets were analyzed in this study. This data can be found here: https://data.mendeley.com/datasets/9424skmnrk/1.
